# Hypercholesterolemic Myocardium Is Vulnerable to Ischemia-Reperfusion Injury and Refractory to Sevoflurane-Induced Protection

**DOI:** 10.1371/journal.pone.0076652

**Published:** 2013-10-04

**Authors:** Yong Xu, Lei-Lei Ma, Chen Zhou, Fei-Jiang Zhang, Fei-Juan Kong, Wen-Na Wang, Ling-Bo Qian, Can-Can Wang, Xian-Bao Liu, Min Yan, Jian-An Wang

**Affiliations:** 1 Department of Anesthesiology, the Second Affiliated Hospital, Zhejiang University School of Medicine, Hangzhou, Zhejaing, China; 2 Department of Anesthesiology, Hangzhou First People’s Hospital, Nanjing Medical University, Hangzhou, Zhejaing, China; 3 Department of Physiology, Zhejiang Medical College, Hangzhou, Zhejaing, China; 4 Department of Cardiology, the Second Affiliated Hospital, Zhejiang University School of Medicine, Hangzhou, Zhejaing, China; Medical College of Wisconsin, United States of America

## Abstract

Recent studies have demonstrated that volatile anesthetic postconditioning confers myocardial protection against ischemia-reperfusion (IR) injury through activation of the reperfusion injury salvage kinase (RISK) pathway. As RISK has been shown to be impaired in hypercholesterolemia. Therefore, we investigate whether anesthetic-induced cardiac protection was maintained in hypercholesterolemic rats. In the present study, normocholesteolemic or hypercholesterolemic rat hearts were subjected to 30 min of ischemia and 2 h of reperfusion. Animals received 2.4% sevoflurane for 5 min or 3 cycles of 10-s ischemia/10-s reperfusion. The hemodynamic parameters, including left ventricular developed pressure, left ventricular end-diastolic pressure and heart rate, were continuously monitored. The infarct size, apoptosis, p-Akt, p-ERK1/2, p-GSK3β were determined. We found that both sevoflurane and ischemic postconditioning significantly improved heart pump function, reduced infarct size and increased the phosphorylation of Akt, ERK1/2 and their downstream target of GSK3β in the healthy rats. In the hypercholesterolemic rats, neither sevoflurane nor ischemic postconditioning improved left ventricular hemodynamics, reduced infarct size and increased the phosphorylated Akt, ERK1/2 and GSK3β. In contrast, GSK inhibitor SB216763 conferred cardioprotection against IR injury in healthy and hypercholesterolemic hearts. In conclusions, hyperchoesterolemia abrogated sevoflurane-induced cardioprotection against IR injury by alteration of upstream signaling of GSK3β and acute GSK inhibition may provide a novel therapeutic strategy to protect hypercholesterolemic hearts against IR injury.

## Introduction

Myocardial ischemia reperfusion injury can be reduced by multiple interventions, such as ischemic postconditioning [1] and volatile anesthetic postconditioning [2], in animal hearts and human hearts. Activation of reperfusion injury salvage kinase (RISK) pathway (mainly PI3K-Akt-GSK3β axis and ERK1/2) contributes to ischemic/anesthetic-induced myocardial protection [1,2]. Multiple prosurvival signaling pathways, including PI3K-Akt and ERK1/2, converge on glycogen synthase kinase 3β (GSK3β) [3]. In addition, the phosphorylation of GSK3β inhibits the opening of mitochondrial permeability transition pore (mPTP) and reduces mitochondria-dependent apoptosis and necrosis [4]. Recent studies have demonstrated that MG53, a newly identified tripartite motif-containing (TRIM) family protein, is indispensable for ischemic postconditioning-induced cardioprotection through the activation of PI3K-Akt-GSK3β axis and ERK1/2 pathway [5]. Although anesthetic and ischemic postconditioning can activate overlapping signal events, whether MG53 is related to the cardiac sevoflurane postconditioning remains elusive.

In recent years, anesthetic postconditioning has mainly been documented in healthy subjects, and the effect of sevoflurane postconditioning on hypercholesterolemic rat heart remains unclear. A number of prospective clinical studies have shown that both coronary artery disease (CAD) and the risk factor for cardiac death after acute myocardial infarct (AMI) are directly related to hypercholesterolemia [6,7]. Furthermore, hypercholesterolemia is associated with alterations of Akt and ERK1/2 phosphorylation and abrogates ischemic postconditioning-induced cardioprotection by interfering with nitric oxide synthase signaling [8,9]. These studies indicate that hypercholesterolemia may adversely affect sevoflurane-induced cardioprotection. Therefore, the aim of the current study was to investigate whether sevoflurane-induced cardioprotection was maintained in hypercholesterolemic rats.

## Materials and Methods

### 1. Animals

All of the animals were treated according to the guidelines of the Guide for the Care and Use of Laboratory Animals (United States National Institutes of Health). The Laboratory Animal Care Committee of Zhejiang University approved all experimental procedures and protocols. All efforts were made to minimize the number of animals used and their suffering. The rats were housed in polypropylene cages, and the room temperature was maintained at 22 °C, with a 12-hour light-dark cycle. Six-week-old male Sprague-Dawley rats, weighing 130-180 g, were used for all experiments.

### 2. Study Groups and Experimental Protocol

To investigate whether sevoflurane-induced cardioprotection was maintained in hypercholesterolemic rats, the experiments were conducted as follows: 1) normocholesterolemic ischemia reperfusion group (IR): rats were fed standard pellet chow for 8 weeks and received no further treatment before myocardial ischemia; 2) normocholesterolemic sevoflurane postconditioning group (IR + SPO): rats were fed standard pellet chow for 8 weeks and then treated with 2.4% sevoflurane (Maruishi Pharmaceutical Co, Ltd, Osaka, Japan) *via* sevoflurane vaporizer (Sevotec 5; Datex-Ohmeda, Tewksbury, MA, USA) for 5-min immediately after reperfusion [10]; 3) normocholesterolemic ischemic postconditioning group (IR + IPO): rats were fed standard pellet chow for 8 weeks and then treated with three episode of 10-s of ischemia/10-s reperfusion immediately after reperfusion [11]; 4) hypercholesterolemic ischemia reperfusion group (HC + IR): rats were fed 2% cholesterol pellet chow for 8 weeks and received no further treatment before myocardial ischemia; 5) hypercholesterolemic sevoflurane postconditioning group (HC + IR + SPO): rats were fed 2% cholesterol pellet chow for 8 weeks and then treated with 2.4% sevoflurane for 5-min immediately after reperfusion. 6) hypercholesterolemic ischemic postconditioning group (HC + IR + IPO): rats were fed standard pellet chow for 8 weeks and then treated with three episode of 10-s of ischemia/10-s reperfusion immediately after reperfusion; To explore the cardioprotective role of GSK3β phosphorylation in hypercholesterolemic rat hearts, we conducted a second experiment. The selective GSK inhibitor SB216763 (0.6 mg/kg i.v., 5 min before reperfusion) [12] were administered to normocholesterolemic and hypercholesterolemic rats. All inhibitors were from Sigma-Aldrich Inc (St Louis, Mo, USA).

### 3. Serum Lipid Assay

Blood was harvested from the caudal vein and centrifuged (3000 rpm, 10 min, 4°C) to obtain serum. Serum lipid levels were measured by spectrophotometry using commercial assay kits for total cholesterol (TC), triglyceride (TG), high-density lipoprotein cholesterol (HDL-C), and low-density lipoprotein cholesterol (LDL-C) (Beijing BHKT Clinical Reagent Co., Ltd., Beijing, China) according to the manuals and as described by Ballantyne et al [13].

### 4. Hematoxylin-eosin Staining

To determine that chronic treatment with a high-cholesterol diet for 8 weeks does not result in the development of coronary atherosclerosis in rats, hematoxylin-eosin staining for thoracic aorta and coronary artery was conducted. The small segments of thoracic aorta and the heart were harvested from rats fed with either high cholesterol or normal chow for 8 weeks for histologic examination. The samples were fixed in 4% paraformaldehyde and embedded in paraffin. Hematoxylin-eosin staining was conducted as Iliodromitis et al [14] have described elsewhere.

### 5. Surgical Preparation

The IR surgery was performed according to the methods of Zhang et al [15] with modifications. Briefly, rats were anesthetized with sodium pentobarbital (50 mg/kg i.p.), plus additional doses (25 mg/kg i.p.) every 60 min to maintain effective anesthesia [16] and were mechanically ventilated with room air to maintain arterial pH, PCO_2_ and PO_2_ within the normal physiological range. The pericardium was opened and a 6-0 silk suture was passed under the left atrial appendage for 2-3 mm through a small polytetrafluoroethylene tube. The tube was pulled to occlude the coronary artery for 30 min and the occlusion was confirmed by epicardial cyanosis in the area at risk, while successful reperfusion (for 120 min) was verified by epicardial hyperemia. Left ventricular developed pressure (LVDP), left ventricular end-diastolic pressure (LVEDP) and heart rate (HR) were measured throughout the experimental period by a polyethylene catheter placed into the left ventricle connecting a pressure transducer connected to a data acquisition system (PowerLab; ADInstruments, Shanghai Trading Co, Ltd, Shanghai, China).

### 6. Determination of Infarct Size

At the end of reperfusion, the coronary artery was reoccluded and perfused with 1% Evans blue dye to identify the unstained area as the area at risk. The left ventricle was separated, frozen, cut into transverse slices, and incubated in 1% triphenyltetrazolium chloride solution at 37°C for 10 min. The area of infarct (pale) and risk (red) was measured by planimetry using ImageJ 1.37 from the National Institutes of Health (Bethesda, MD, USA). The infarct size was expressed as a percentage of the area at risk. Samples with an area at risk <15% or >45% of the left ventricle were excluded [17].

### 7. Detection of Myocardial Apoptosis

Apoptosis was assessed using the TUNEL method. At the end of reperfusion, the hearts were fixed in 4% paraformaldehyde and embedded in paraffin for TUNEL staining. The heart tissue sections were stained using an in situ cell death detection kit (POD; Roche Diagnostics Corp, Indianapolis, IN, USA), following the manufacturer’s protocol. Ten microscopic fields (400×) from each section were assayed by counting brown nuclei. The percentage of TUNEL-positive nuclei (brown nuclei) was calculated.

### 8. Immunoblotting

Fifteen minutes following reperfusion, the samples were taken from ischemic zone. The expression of myocardial Akt, phosphorylated-Akt (Ser^473^) (p-Akt), ERK1/2 and phosphorylated-ERK1/2 (Thr^202^/Tyr^204^) (p-ERK1/2), GSK3β, phosphorylated-GSK-3β (Ser^9^) (p-GSK3β) (Cell Signaling Technology, Beverly, MA, USA) were determined by Western blotting as we described elsewhere [18]. At the end of reperfusion, the expression of MG53 (Gene Tex, San Antonio, TX, USA) and PI3K-p85 (Cell Signaling Technology, Beverly, MA, USA) was determined by Western blotting as we described elsewhere [18]. The quantitative protein band density was assayed by ImageJ 1.37.

### 9. Statistical Analysis

Data are shown as mean ± SD. Lipid levels were analyzed using the unpaired Student’s *t* test. All other data were analyzed by one-way ANOVA following Newman-Keuls *post hoc* test. A value of *P* <0.05 was considered to be statistically significant. All statistical analyses were performed using GraphPad Prism Version 4.0 (GraphPad Prism Software, San Diego, CA, USA).

## Results

Seventy rats were used for myocardial infarction experiments (2 were excluded as a result of area at risk <15% and 4 died of refractory ventricular arrhythmias during the 30-min occlusion) and 79 animals were used for immunoblotting (7 died of refractory ventricular arrhythmias during the 30-min occlusion). An additional 53 rats were used for TUNEL staining (5 died of refractory ventricular arrhythmias during the 30-min occlusion). 10 rats were used for hematoxylin-eosin staining.

### 1. High-cholesterol Diet Induced a Moderate and Steady Increase in Serum Cholesterol without Substantial Development of Coronary Atherosclerosis in rats

Levels of TC (136.5 ± 12.8 mg/dl), LDL-C (57.9 ± 7.2 mg/dl), and TG (128.2 ± 7.1 mg/dl) were increased in rats fed high-cholesterol chow compared with those (68.3 ± 6.6 mg/dl, 26.1 ± 4.8 mg/dl, and 59.4 ± 8.9 mg/dl) fed normal chow (*P* < 0.01). The level of HDL-C (29.6 ± 3.9 *vs.* 28.3 ± 4.8) was not significantly different between normocholesterolemic and hypercholesterolemic rats.

**Figure 1:**
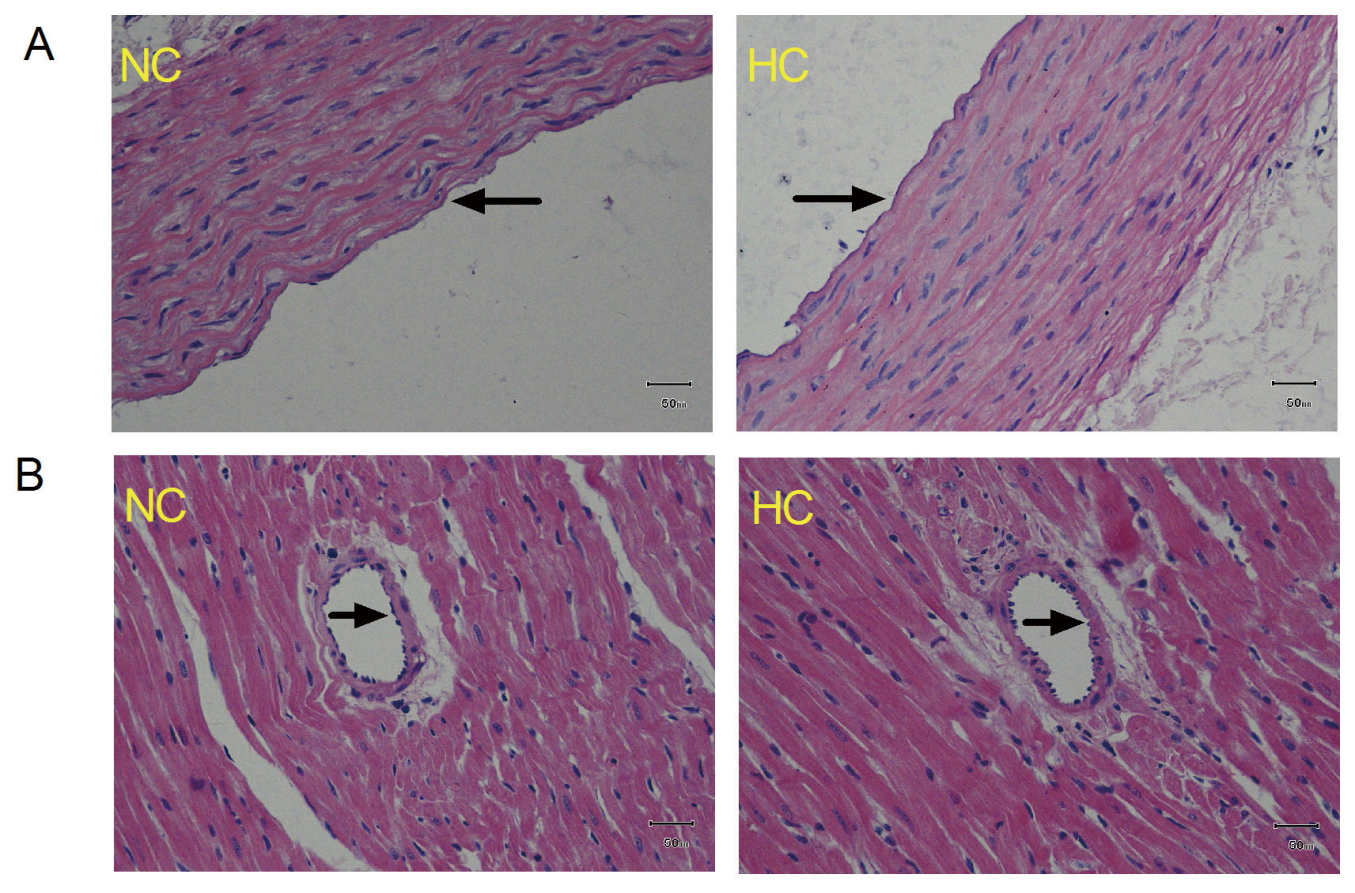
Representative cross-sections of the thoracic aorta (A) and the left myocardial branch of the coronary artery (B) from normocholesterolemic (NC) and hypercholesterolemic (HC) rats (fed 2% cholesterol-enriched chow for 8 weeks) by hematoxylin-eosin (HE) staining (400×). The arterial lumen is indicated by the arrow, n = 5 hearts/group.

Atherogenesis was detected in the form of subintimal accumulation of lipids and foamy macrophages. There was no deposition of lipids and foamy macrophages in the subintimal area in the cross-section of the aortic wall (Figure 1A) and the myocardial branch of coronary artery from rats fed with either high cholesterol or normal chow for 8 weeks (Figure 1B).

### 2. Hypercholesterolemia Abrogates the Left Ventricular Hemodynamic Improvement Induced by Sevoflurane and Ischemic Postconditioning

As shown in Table 1, there was no significant difference in baseline left ventricular hemodynamic parameters among all groups (*P* > 0.05). After 60 min of reperfusion, LVDP and LVEDP were significantly increased in IR + SPO and IR + IPO compared with that in IR (*P* < 0.05). However, such improving effect was not observed in HC + SPO and HC + IPO compared with that in HC + IR (*P* > 0.05).

**Table 1:** The effect of sevoflurane and ischemic postconditioning on left ventricular hemodynamic parameters in rat hearts exposed to ischemia-reperfusion.

Group	Baseline	Ischemia	Reperfusion			
			30 min	60 min	90 min	120 min
LVDP, mmHg						
IR	127 ± 8	81 ± 5	108 ± 11	89 ± 9	84 ± 6	77 ± 10
IR + SPO	130 ± 9	83 ± 5	111 ± 11	106 ± 5*	101 ± 5*	97± 6*
IR + IPO	123 ± 7	80 ± 7	115 ± 15	107 ± 11*	102 ± 5*	96 ± 7*
HC + IR	130 ± 12	85 ± 11	115 ±10	98 ± 9	88 ± 12	80 ± 13
HC + IR + SPO	134 ± 8	80 ± 7	110 ± 7	94 ± 12	87 ± 13	83 ± 12
HC + IR + IPO	127 ± 7	84 ± 5	107 ± 10	92 ± 7	86 ± 7	84 ± 8
LVEDP, mmHg						
IR	4.1 ± 1.5	10.1 ± 1.6	5.3 ± 1.8	7.6 ± 1.8	7.9 ± 1.4	8.2 ± 1.5
IR + SPO	3.9 ± 1.0	9.4 ± 1.7	4.5 ± 1.4	5.1 ± 1.2*	5.9 ± 1.1*	5.7 ± 1.5*
IR + IPO	4.0 ± 1.4	9.5 ± 2.1	5.0 ± 1.1	5.6 ± 1.2*	5.5 ± 1.0*	5.9 ± 1.1*
HC + IR	4.1 ± 1.0	9.9 ± 1.2	5.4 ± 1.0	6.9 ± 1.3	7.8 ± 1.0	8.4 ± 1.3
HC + IR + SPO	4.5 ± 1.2	10.4 ± 1.1	6.3 ± 1.0	7.2 ± 1.2	7.4 ± 1.2	7.8 ± 1.5
HC + IR + IPO	3.9 ± 1.4	10.0 ± 1.8	6.4 ± 1.6	7.0 ± 1.8	7.3 ± 1.5	7.7 ± 1.6
HR, beats/min						
IR	345 ± 35	325 ± 41	312 ± 48	305 ± 39	315 ± 42	299 ± 41
IR + SPO	332 ± 32	312 ± 41	315 ± 39	321 ± 35	306 ± 40	313 ± 39
IR + IPO	350 ± 34	310 ± 34	318 ± 40	309 ± 37	310 ± 39	300 ± 41
HC + IR	328 ± 31	308 ± 43	303 ± 39	300 ± 34	298 ± 41	288 ± 39
HC + IR + SPO	340 ± 39	318 ± 39	300 ± 39	310 ± 30	313 ± 44	304 ± 41
HC + IR + IPO	330 ± 38	320 ± 38	310 ± 32	308 ± 36	310 ± 33	300 ± 37

Effects of sevoflurane and ischemic postconditioning on left ventricular hemodynamic parameters in rat hearts exposed to ischemia-reperfusion. IR: ischemia reperfusion; SPO: sevoflurane posconditioning; IPO: ischemic posconditioning; HC: hypercholesterolemia. Data are mean ± SD, n = 8 hearts/group. *P < 0.05 vs. IR.

### 3. Hypercholesterolemia Abrogates the Myocardial Infarct-sparing Effect of Sevoflurane and Ischemic Postconditioning

**Figure 2:**
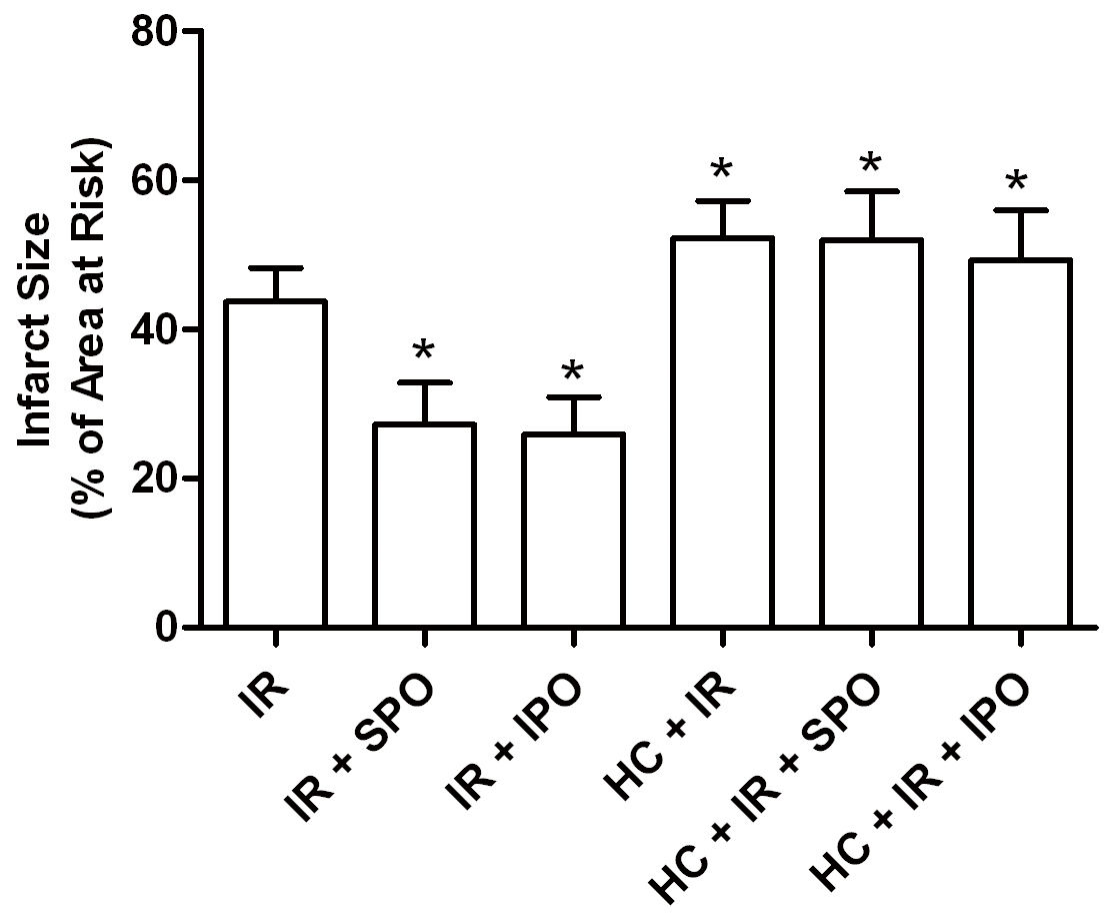
Effects of sevoflurane and ischemic postconditioning on infarct size expressed as a percentage of area at risk in rat hearts exposed to ischemia-reperfusion. IR: ischemia reperfusion; SPO: sevoflurane posconditioning; IPO: ischemic posconditioning; HC: hypercholesterolemia. Data are mean ± SD, n = 8 hearts/group. *P < 0.05 *vs*. IR.

The area at risk was not significantly different among all groups (data not shown). As shown in Figure 2, the infarct size was larger by 18% in the HC + IR (52 ± 5%) compared with the IR group (44 ± 5%, *P* < 0.05). The infarct size was markedly decreased in the IR + SPO (27 ± 6%) and IR + IPO (26 ± 5%) over the IR group (44 ± 5%, *P* < 0.05). The reduced myocardial infarct conferred by sevoflurane and ischemic postconditioning was not found in hypercholesterolemic rats.

### 4. Hypercholesterolemia Inhibits the Myocardial Anti-apoptotic Effect of Sevoflurane and Ischemic Postconditioning

**Figure 3:**
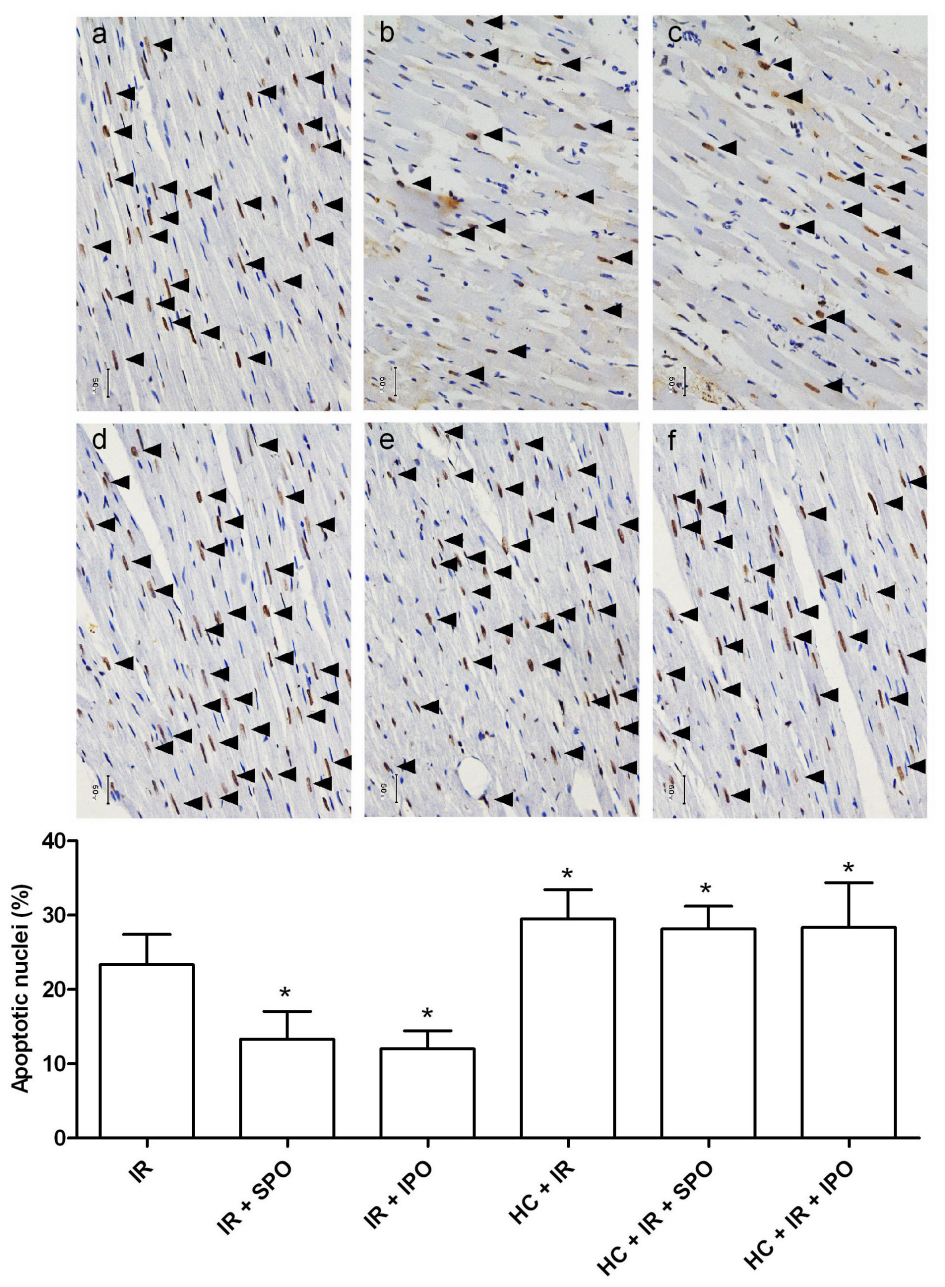
Effects of sevoflurane and ischemic postconditioning on apoptosis expressed as a percent of total nuclei in tissue sections from rat hearts exposed to ischemia-reperfusion (TUNEL staining, 400×). TUNEL-positive nuclei (brown nuclei) was indicated by the arrow. a normocholesterolemic ischemia reperfusion group (IR), b normocholesterolemic sevoflurane postconditioning group (IR + SPO), c normocholesterolemic ischemic postconditioning group (IR + IPO), d hypercholesterolemic ischemia reperfusion group (HC + IR), e hypercholesterolemic sevoflurane postconditioning group (HC + IR + SPO), f hypercholesterolemic ischemic postconditioning group (HC + IR + IPO). IR: ischemia reperfusion; SPO: sevoflurane postconditioning; IPO: ischemic postconditioning; HC: hypercholesterolemia. Data are mean ± SD, n = 6 hearts/group. *P < 0.05 *vs*. IR.

As shown in Figure 3, The number of TUNEL-positive nuclei expressed as a percentage of total nuclei was markedly decreased in the IR + SPO (13 ± 4%) and IR + IPO (12 ± 2%) over the IR group (23 ± 4%, *P* < 0.05), however, the reduced apoptotic nuclear conferred by sevoflurane and ischemic postconditioning was not found in hypercholesterolemic rats. Interestingly, the number of TUNEL-positive nuclei was increased by 30% in the HC + IR (30 ± 4%) compared to the IR group (23 ± 4%, *P* < 0.05).

### 5. Hypercholesterolemia Abrogates the Upregulation of MG53 Expression Induced by Sevoflurane and Ischemic Postconditioning

Neither sevoflurane nor hypercholesterolemia alters the expression of MG53 in the absence of IR, which indicates that sevoflurane doesn’t have a direct effect on MG53 expression (Figure S1).

**Figure 4:**
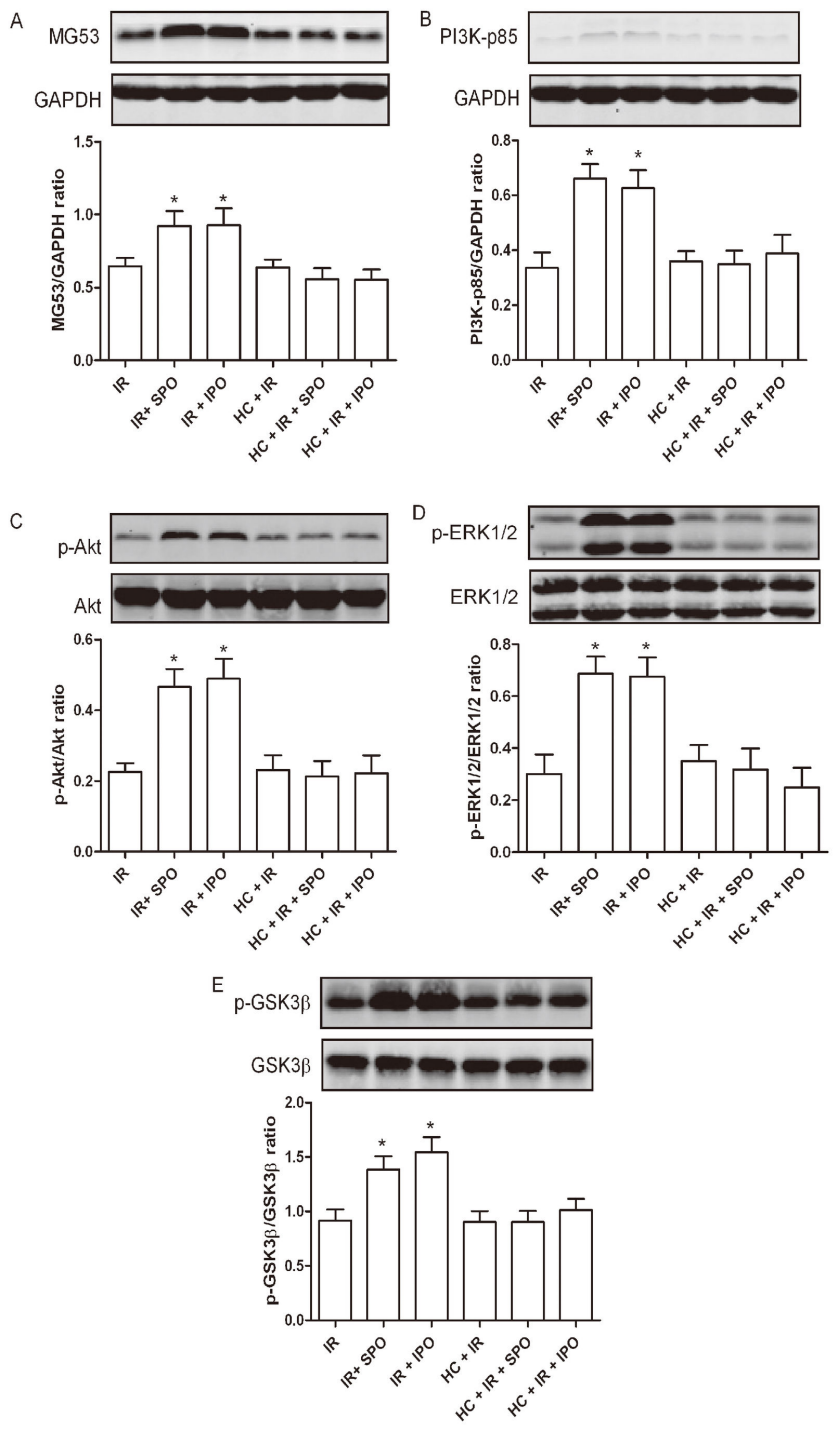
Effects of sevoflurane and ischemic postconditioning on the expression of MG53 (A), PI3K-p85 (B), p-Akt (C), p-ERK1/2 (D), p-GSK3β (E) in rat hearts exposed to ischemia-reperfusion (IR). IR: ischemia reperfusion; SPO: sevoflurane postconditioning; IPO: ischemic postconditioning; HC: hypercholesterolemia. Data are mean ± SD, n = 6 hearts/group. *P < 0.05 *vs*. IR.

Sevoflurane and ischemic postconditioning upregulated the expression of MG53 in healthy rats (*P* < 0.05), but this was completely blocked by hypercholesterolemia (Figure 4A). The expression of MG53 was not significantly different between IR and HC + IR groups.

### 6. Hypercholesterolemia Abrogates the Upregulation of PI3K-p85 and p-Akt Expression Induced by Sevoflurane and Ischemic Postconditioning

The levels of total Akt were not significantly different among all groups. Therefore, the levels of p-Akt were expressed as the percentage of total protein. Sevoflurane and ischemic postconditioning significantly increased the expression of PI3K-p85 and p-Akt in healthy rats (*P* < 0.05), however, this didn’t occur in hypercholesterolemic ones. The expression of PI3K-p85 and p-Akt didn’t significantly differ between IR and HC + IR groups (Figure 4B and Figure 4C).

### 7. Hypercholesterolemia Abrogates the Upregulation of p-ERK1/2 Expression Induced by Sevoflurane and Ischemic Postconditioning

No significant differences were found in the expression of total ERK1/2 among any groups. Sevoflurane and ischemic postconditioning significantly increased the p-ERK1/2 in healthy rats (*P* < 0.05). Interestingly, this effect was inhibited in the hypercholesterolemic groups. No difference in the expression of p-ERK1/2 was observed between IR and HC + IR groups (Figure 4D).

### 8. Hypercholesterolemia Abrogates the Upregulation of p-GSK3β Expression Induced by Sevoflurane and Ischemic Postconditioning

Immunoblots of total GSK3β were not significantly different between groups. Sevoflurane and ischemic postconditioning significantly increased the p-GSK3β in healthy rats (*P* < 0.01), however, this effect was blocked by hypercholesterolemia. The expression of p-GSK3β didn’t significantly differ between IR and HC + IR groups (Figure 4E).

### 9. GSK3β Inhibitor was Cardioprotective in Hypercholesterolemic Hearts

To investigate whether the significant inhibition of sevoflurane and ischemia-induced GSK3β phosphorylation in hypercholesterolemic rat hearts was due to the inactivation of Akt and ERK1/2, we treated healthy and hypercholesterolmic rats with GSK3β inhibitor SB216763.

While sevoflurane and ischemic postconditioning did not improve the hemodynamic parameters of hypercholesterolemic rat hearts, SB216763 significantly improved LVDP and LVEDP in the hypercholeslerolemic group as well as in the healthy group after 60 min of reperfusion (Table 2).

**Table 2:** The effect of GSK3β inhibitor SB216763 on left ventricular hemodynamic parameters in rat hearts exposed to ischemia-reperfusion.

Group	Baseline	Ischemia	Reperfusion			
			30 min	60 min	90 min	120 min
LVDP, mmHg						
IR	127 ± 8	81 ± 5	108 ± 11	89 ± 9	84 ± 6	77 ± 10
IR + SB	125 ± 9	83 ± 9	110 ± 12	105 ± 8*	102 ± 7*	95 ± 7*
HC + IR	130 ± 12	85 ± 11	115 ±10	98 ± 9	88 ± 12	80 ± 13
HC + IR + SB	127 ± 7	84 ± 8	117 ± 10	110 ± 7^#^	100 ± 7^#^	93 ± 8^#^
LVEDP, mmHg						
IR	4.1 ± 1.5	10.1 ± 1.6	5.3 ± 1.8	7.6 ± 1.8	7.9 ± 1.4	8.2 ± 1.5
IR + SB	3.8 ± 1.1	9.8 ± 1.5	5.0 ± 1.4	5.3 ± 1.2*	5.7 ± 1.3*	5.9 ± 1.2*
HC + IR	4.1 ± 1.0	9.9 ± 1.2	5.4 ± 1.0	6.9 ± 1.3	7.8 ± 1.0	8.4 ± 1.3
HC + IR + SB	3.9 ± 1.4	9.6 ± 1.4	4.9 ± 1.2	5.6 ± 1.1	5.8 ± 1.3^#^	6.0 ± 1.4^#^
HR, beats/min						
IR	345 ± 35	325 ± 41	312 ± 48	305 ± 39	315 ± 42	299 ± 41
IR + SB	322 ± 33	310 ± 36	320 ± 33	311 ± 39	315 ± 36	310 ± 33
HC + IR	328 ± 31	308 ± 43	303 ± 39	300 ± 34	298 ± 41	288 ± 39
HC + IR + SB	320 ± 38	310 ± 39	312 ± 30	318± 41	310 ± 37	302 ± 34

Effects of GSK3β inhibitor SB216763 on left ventricular hemodynamic parameters in rat hearts exposed to ischemia-reperfusion. IR: ischemia reperfusion; SB: SB216763; HC: hypercholesterolemia. Data are mean ± SD, n = 8 hearts/group. *P < 0.05 *vs.* IR, ^#^ *P* < 0.05 *vs.* HC + IR.

**Figure 5:**
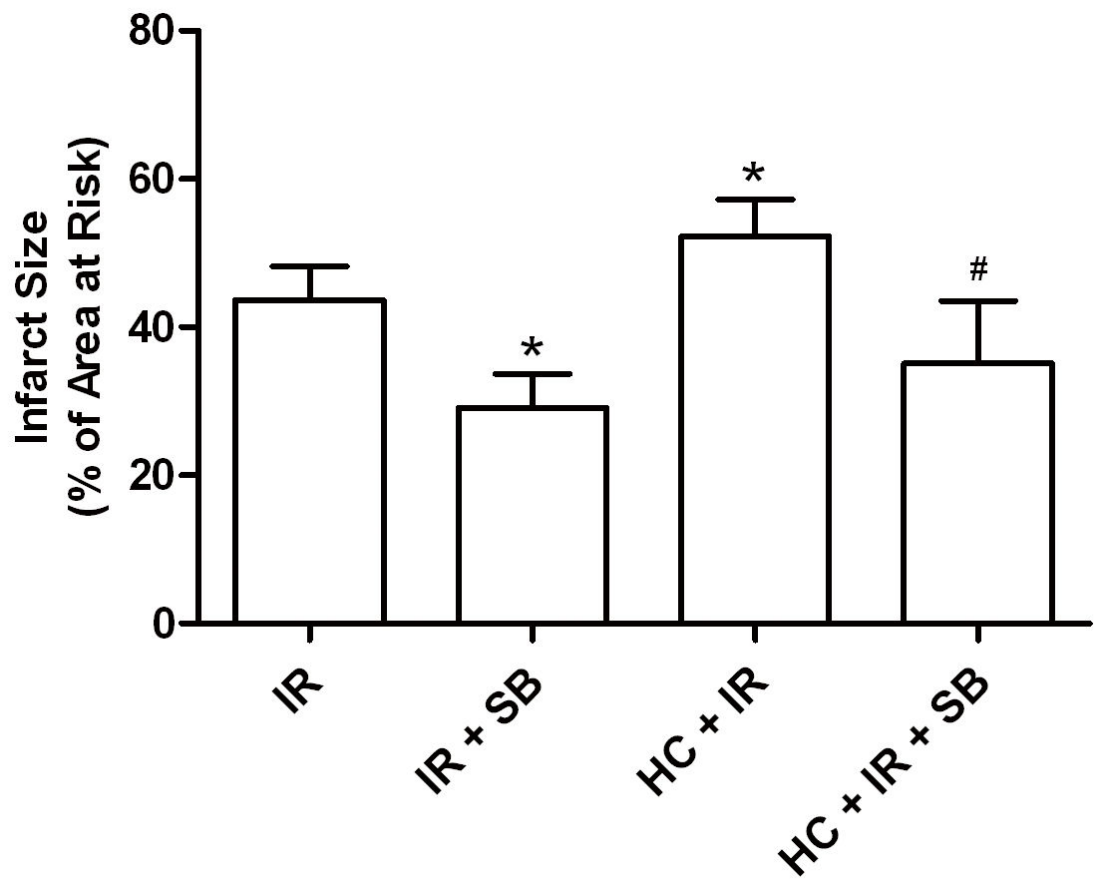
Effects of GSK3β inhibitor SB216763 on infarct size expressed as a percentage of area at risk in rat hearts exposed to ischemia-reperfusion. IR: ischemia reperfusion; SB: SB216763; HC: hypercholesterolemia. Data are mean ± SD, n = 8 hearts/group. *P < 0.05 *vs*. IR; ^#^ *P* < 0.05 *vs*. HC + IR.

Interestingly, we found that SB216763 significantly reduced infarct size in both normal and hypercholesterolemic hearts (IR + SB, 29 ± 5% *vs.* IR, 44 ± 5%; HC+ IR + SB, 35 ± 8% *vs.* HC + IR, 52 ± 5%; *P* < 0.05) (Figure 5).

**Figure 6:**
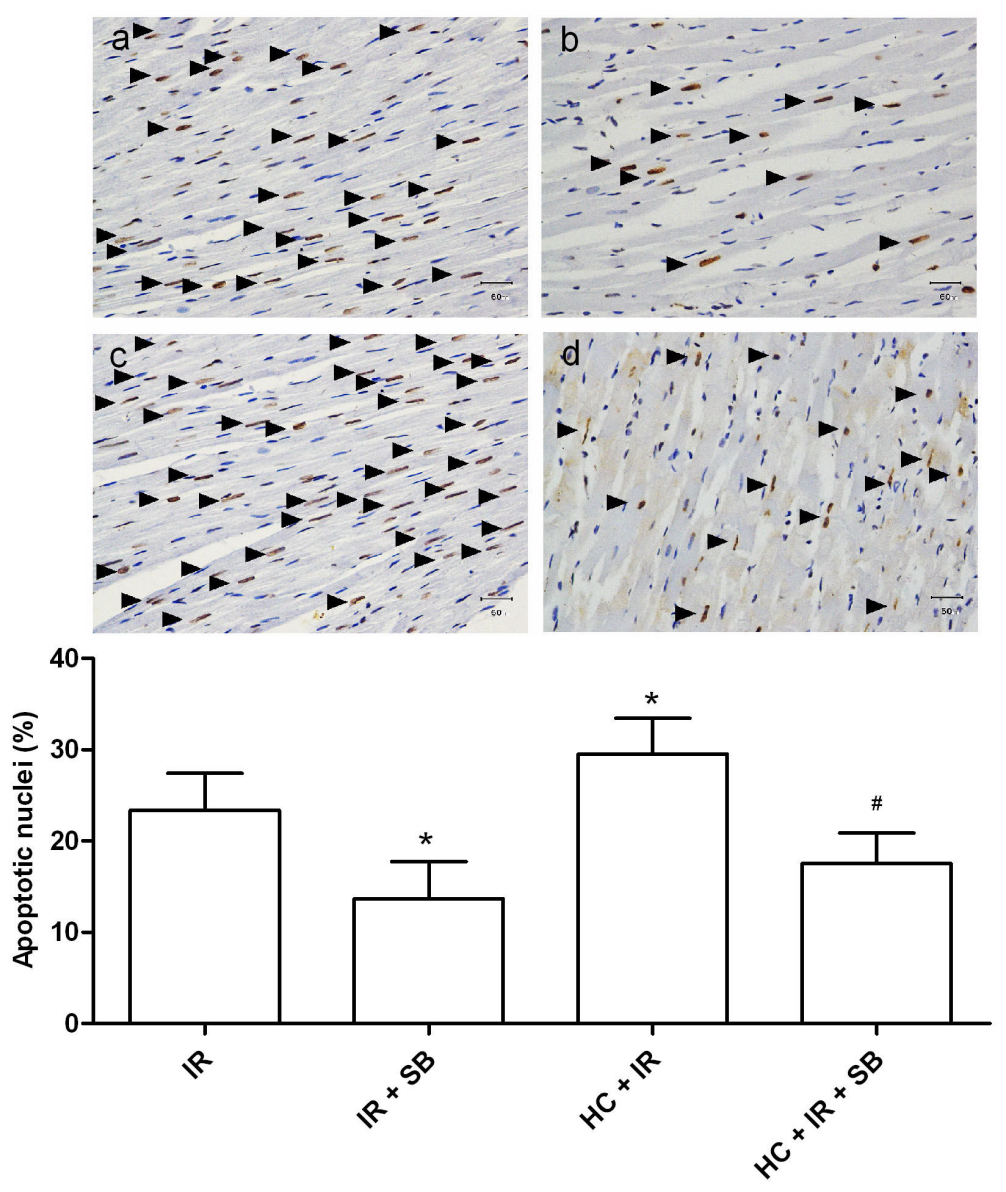
Effects of GSK3β inhibitor SB216763 on apoptosis expressed as a percent of total nuclei in tissue sections from rat hearts exposed to ischemia-reperfusion (TUNEL staining, 400×). a normocholesterolemic ischemia reperfusion group (IR), b normocholesterolemic SB216763-treated group (IR + SB), c hypercholesterolemic ischemia reperfusion group (HC + IR), d hypercholesterolemic SB216763-treated group (HC + IR + SB). IR: ischemia reperfusion; SB: SB216763; HC: hypercholesterolemia. Data are mean ± SD, n = 6 hearts/group. *P < 0.05 *vs*. IR; ^#^ *P* < 0.05 *vs*. HC + IR.

Moreover, SB216763 significantly decreased the number of TUNEL-positive nuclei in both healthy and hypercholesterolemic hearts (IR + SB, 14 ± 4% *vs.* IR, 23 ± 4%; HC+ IR + SB, 18 ± 3% *vs.* HC + IR, 30 ± 4%; *P* < 0.05) (Figure 6).

## Discussion

The current study suggested that sevoflurane and ischemic postconditioning-induced cardioprotection against IR was blunted in hypercholesterolemic rat hearts, with altered MG53/RISK signaling pathway that inhibit GSK3β. We found that inhibition of GSK3β may be a potential therapeutic intervention to reduce myocardial infarct and apoptosis in hypercholesterolemic subjects.

We used chronic treatment with a high-cholesterol diet for 8 weeks to induce hypercholesterolemia in rats [19,20]. We found that this diet induced a moderate and steady increase in serum cholesterol without substantial development of coronary atherosclerosis (Figure 1), which is consistent with previous studies and suggests that the factor influencing sevoflurane postconditioning in our study was hypercholesterolemia itself, and not subsequent atherosclerosis or other hypercholesterolemic complications [21,22]. Therefore, we used this hypercholesterolemic model to study the direct cardiac effects of hypercholesterolemia on sevoflurane postconditioning.

Effects of hypercholesterolemia on myocardial IR have yielded controversial results in rodents. A number of studies have shown that hypercholesterolemia increases myocardial infarct size in the setting of IR [8,19,20], but the underlying mechanism is poorly understood. In our study, the myocardial infarct size induced by 30-min occlusion and 120-min reperfusion was increased by 18% in hypercholesterolemic rat hearts than that in healthy ones and suggests that hypercholesterolemic rats are susceptible to IR injury. In contrast to our findings, Kupai et al [23] showed that the myocardial infarct size induced by 30-min left coronary artery occlusion and 120-min reperfusion was not significantly increased in hypercholesterolemic rats fed a 2% cholesterol-enriched diet for 12 weeks compared with that in normally fed rats, and Giricz et al [24] reported that the infarct size induced by 30-min global ischemia and 120-min reperfusion in hearts isolated from rats fed a 2% cholesterol diet for 12 weeks was not significantly increased than that from normally fed rats. The reasons for such diverse results are unknown but may be attributable to the different high-cholesterol diet treatment periods and the different IR models (in vivo or in vitro) [8,19,20,23,24].

Another principle finding of our current study was hypercholesterolemia alone increased myocardial apoptosis in the setting of IR. In the present study, the number of apoptotic nuclei was increased by 30% in hypercholesterolemic rat hearts than that in healthy ones. One reasonable explanation is increased free oxygen radicals and inflammation may prompt apoptotic signaling pathway [25]. Furthermore, hypercholesterolemia is associated with decreased expression of anti-apoptotic Bcl-2 [8], which plays a pivotal role in preventing apoptosis by inhibiting the activation of executioner caspases 3 [26] and the release of mitochondrial cytochrome c [27]. All of the above may contribute to the increased myocardial apoptosis in the setting of hypercholesterolemia during IR.

The present study has shown that hypercholesterolemia induced by high-cholesterol diet abrogated sevoflurane and ischemic postconditioning-induced cardioprotection and modified cardioprotective signaling pathways. Here, we demonstrated that the myocardial p-Akt and p-ERK1/2 expression was upregulated by sevoflurane and ischemic postconditioning. Interestingly, such cardioprotection and upregulation of myocardial p-Akt and p-ERK1/2 expression were both lost in hypercholesterolemic rats. Furthermore, previous studies have demonstrated that both PI3k-Akt and MEK-ERK1/2 signals play a pivotal role in the cardioprotection of sevoflurane or ischemic postconditioning [1,2]. These results at least indicate that the loss of upregulation of p-Akt and p-ERK1/2 expression is closely associated with the loss of cardioprotection of sevoflurane postconditioning in hypercholesterolemic rats. MG53, an upstream signaling cascade of Akt, forms a functional complex with the p85 subunit of PI3K and contributes to acute membrane repair in cardiomyocytes [5,28]. In addition, ischemic postconditioning-induced cardioprotection is lost in MG53-deficient mice and overexpression of MG53 attenuates hypoxia- and oxidative-induced cardiomyocytes death through the activation of PI3K-Akt-GSK3β and ERK1/2 signaling pathways [5,29]. In healthy rats, sevoflurane significantly increased the levels of MG53 and PI3k-p85, however, this didn’t occur in hypercholesterolemic ones with sevoflurane postconditioning. Our results indicate that the dysfunction of the upstream protein kinases MG53 may in part contribute to the observed deficit in Akt and ERK1/2 phosphorylation in hypercholesterolemic rat hearts. Furthermore, the deficit in GSK3β phophorylation in hypercholesterolemic rat hearts may in part be attributed to the observed deficit in Akt and ERK1/2 phosphorylation. If, as mentioned above, upregulation of phosphorylated Akt, ERK1/2 and GSK3β plays a pivotal role in the infarct-sparing effect of sevoflurane postconditioning, we reasoned that the loss of cardioprotection in hypercholesterolemic myocardium, at least in part, be attributed to the dysfunction of Akt, ERK1/2 and GSK3β phosphorylation.

Our current study has shown that the cardioprotective effect of postconditioning was lost in hypercholesterolemic subjects, which suggests that hypercholesterolemic patients may not benefit from therapeutic application of sevoflurane in the clinical setting. Therefore, it is translationally important to explore novel cardioprotective strategies. We found that the GSK3β inhibitor SB216763 significantly improved heart pump function, reduced myocardial infarct size and apoptosis in hypercholesterolemic rats, which suggests that the downstream signal mechanisms of GSK3β in hypercholesterolemic myocardium is preserved. Together, these data indicate that inhibition of GSK3β would be a more promising therapeutic target to protect hypercholesterolemic hearts against IR injury.

Our findings are translationally important in that they determined whether the myocardial protection induced by sevoflurane occurs in hypercholesterolemic animals. However, there are several limitations in our current study. First, the loss of cardioprotection of sevoflurane postconditioning in hypercholesterolemic rats might also be due to changes in cardiovascular oxidative/nitrosative stress [23,30], which were not explored in the current study. Second, patients with hypercholesterolemia are usually also have coronary atherosclerosis, and our hypercholesterolemic rats did not, which indicates that the hypercholesterolemic rat model we used may not fully simulate the complex clinical setting of hypercholesterolemic patients, so our results may only apply to effect of hypercholesterolemia on cardioprotection induced by sevoflurane in a limited clinical setting.

In summary, we report that sevoflurane-induced cardioprotection against IR injury was abrogated in hypercholesterolemic rats. Hypercholesterolemia blocked the ability of sevoflurane to phosphorylate components of RISK pathway and consequently, the phosphorylation of the downstream target GSK3β. These data indicate that direct inhibition of GSK3β before myocardial infarct may be a potential therapeutic approach to prevent IR injury in the presence of hypercholesterolemia.

## Supporting Information

Figure S1 **Effects of sevoflurane on the expression of MG53 in sham-operated rat hearts.** Normocholesterolemic and hypercholesterolemic sham-operated rats were treated with 2.4% sevoflurane *via* sevoflurane vaporizer for 5-min. Then hearts were harvested for immunoblotting. NC: normocholesterolemia; HC: hypercholesterolemia. Sev: sevoflurane. Data are mean ± SD, n = 6 hearts/group.
(TIF)
