# Supplementary figures and images for: Hypercholesterolemic Myocardium Is Vulnerable to Ischemia-Reperfusion Injury and Refractory to Sevoflurane-Induced Protection
Source: PLoS One. 2013 Oct 4;8(10):e76652. doi: 10.1371/journal.pone.0076652 (PMC3790738; doi:10.1371/journal.pone.0076652)

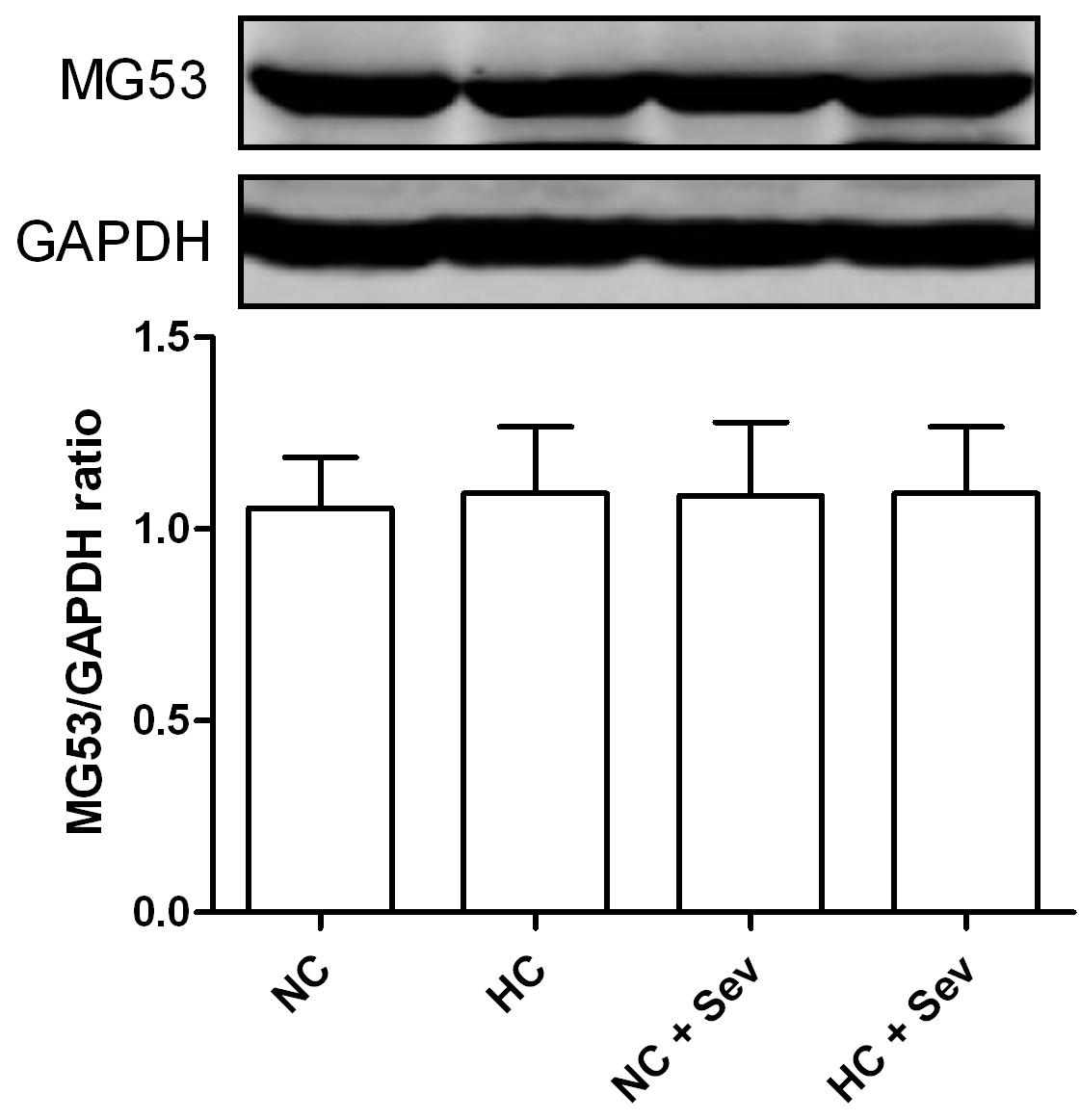

Supplement: Figure S1 — Effects of sevoflurane on the expression of MG53 in sham-operated rat hearts. Normocholesterolemic and hypercholesterolemic sham-operated rats were treated with 2.4% sevoflurane via sevoflurane vaporizer for 5-min. Then hearts were harvested for immunoblotting. NC: normocholesterolemia; HC: hypercholesterolemia. Sev: sevoflurane. Data are mean ± SD, n = 6 hearts/group. (TIF) [file pone.0076652.s001.tif]
